# New Life-preserver

**Published:** 1844-02

**Authors:** 


### A NEW LIFE-PRESERVER.

Our friend, Dr. L. H. Mosby, has invented a *life-preserver* which strikes us as superior to anything of the sort ever yet proposed. It consists of a tin box rendered air-tight, which is to be fitted to the bottoms of the chairs on steam boats, and which, without being in the way, will form a buoy, always at hand, upon which several persons might float in case of accident. It is at once cheap, simple and efficient, and we have no doubt, will soon be found in use on all our steam boats. Dr. Mosby has taken out a patent for it. Y.

